# The Impact of the COVID-19 Pandemic on the Lifestyles and Levels of Anxiety and Depression of Patients with Schizophrenia: A Retrospective Observational Study

**DOI:** 10.3390/healthcare10010128

**Published:** 2022-01-09

**Authors:** Gemma Biviá-Roig, Pau Soldevila-Matías, Gonzalo Haro, Victor González-Ayuso, Francisco Arnau, Loreto Peyró-Gregori, Laura García-Garcés, Maria I. Sánchez-López, Juan Francisco Lisón

**Affiliations:** 1 Department of Nursing and Physiotherapy, Faculty of Health Sciences, University CEU-Cardenal Herrera, CEU Universities, 46115 Valencia, Spain; lpeyro@uchceu.es (L.P.-G.); laura.garcia19@uchceu.es (L.G.-G.); maria.sanchez6@uchceu.es (M.I.S.-L.); 2 State Reference Center for Psychosocial Rehabilitation (Creap), 46015 Valencia, Spain; pausoldevila.externo@imserso.es (P.S.-M.); victorgonzalez.externo@imserso.es (V.G.-A.); 3 Department of Basic Psychology, Faculty of Psychology, University of Valencia, 46015 Valencia, Spain; 4 TXP Research Group, Medicine & Surgery Department, Universidad Cardenal Herrera-CEU, CEU Universities, 46115 Valencia, Spain; gonzalo.haro@uchceu.es; 5 Mental Health Department, Consorcio Hospitalario Provincial de Castellón, 12002 Castellón, Spain; thot20240@hotmail.com; 6 Department of Biomedical Sciences, Faculty of Health Sciences, University CEU-Cardenal Herrera, CEU Universities, 46115 Valencia, Spain; juanfran@uchceu.es; 7 CIBER of Physiopathology of Obesity and Nutrition CIBERobn, CB06/03 Carlos III Health Institute, 28029 Madrid, Spain

**Keywords:** COVID-19, schizophrenia, anxiety, depression, diet, lifestyle, mental disorders

## Abstract

The movement restrictions put in place as a result of the COVID-19 pandemic required modification of the population’s usual routines, including those of the most vulnerable groups such as patients with schizophrenia. This was a retrospective observational study. We used an online survey to collect information on patient adherence to the Mediterranean diet (Mediterranean Diet Adherence Screener questionnaire), physical exercise (International Physical Activity Questionnaire Short Form), and tobacco consumption and levels of anxiety and depression (Hospital Anxiety and Depression Scale) before and during the movement restrictions. A total of 102 people with schizophrenia participated in this study. During the COVID-19 pandemic lockdown the participants significantly increased the number of minutes spent sitting per day (z = −6.73; *p* < 0.001), decreased the time they spent walking (z = −6.32; *p* < 0.001), and increased their tobacco consumption (X^2^ = 156.90; *p* < 0.001). These results were also accompanied by a significant increase in their reported levels of anxiety (z = −7.45; *p* < 0.001) and depression (z = −7.03, *p* < 0.001). No significant differences in patient diets during the pandemic compared to before the movement restrictions were reported. These results suggest the need to implement specific programs to improve lifestyle and reduce anxiety and depression during possible future pandemic situations.

## 1. Introduction

Since the initial emergence of the SARS-CoV-2 virus in Wuhan (China), the COVID-19 disease caused by this new coronavirus spread exponentially to rapidly become a global pandemic. To reduce the number of COVID-19 infections, the governments of different countries imposed social isolation and confinement measures, with the consequent drastic modification of the daily routines of the population. Given the importance of lifestyle on health, the World Health Organization (WHO) recommends maintaining a healthy diet and routinely engaging in physical exercise [1]. However, these population-wide public health recommendations may be less effective for certain more vulnerable groups, including patients with severe mental disorders such as schizophrenia [2].

The incidence rate in schizophrenia is 21.4 per 100,000 person-years, and the average age when the first episode occurs is 30.5 years [3]. It is a chronic disorder characterised by the presence of positive symptoms (hallucinations, delusions, and thought disorders) and negative symptoms (withdrawal, apathy, and social dysfunction, among others). The cognitive impairments presented by these patient types makes these conditions one of the main causes of disability, with serious personal, social, and economic consequences [4,5,6].

Patients with schizophrenia often have unhealthy lifestyles characterised by sedentary habits, an inadequate diet, and increased tobacco consumption. These factors also increase the risk of developing other pathologies such as obesity, hypertension, or diabetes [7,8]. Factors predisposing to an unhealthy lifestyle in these individuals include educational level, socioeconomic status, and social isolation [9]. In addition, cognitive symptoms such as a lack of motivation, apathy, cognitive deficits, and even the side effects of antipsychotic drugs can also become obstacles to the development of healthy lifestyles [10,11]. The use of antipsychotic drugs is also related to problems with increase in appetite, weight gain, obesity and associated metabolic syndromes such as hyperglycaemia or type II diabetes [12,13,14]. It is also believed that physical activity in these patients may be influenced by drug-induced extrapyramidal symptoms [15]. All this means that patients with schizophrenia present higher rates of comorbidities and a higher incidence of mortality compared to the general population [10,16].

Importantly, in the new context of the COVID-19 pandemic, the social isolation measures and movement restrictions imposed to contain the SARS-CoV-2 virus may have had a negative impact on the lifestyles of this population group. Most of the studies published to date in patients with schizophrenia have focused mainly on the effects of the pandemic on different psychological aspects such as anxiety and depression, psychotic symptoms or coping strategies [17,18,19]. To our knowledge, however, there are not previous studies that have analysed the lifestyle of this kind of patients during the COVID-19 pandemic. Therefore, the objective of this study was to analyse the impact of movement restrictions resulting from the COVID-19 pandemic on the diets, exercise levels, smoking habits, and levels of anxiety and depression among patients with schizophrenia.

## 2. Materials and Methods

This was a retrospective observational study which used an anonymous survey. The questionnaire was distributed via a Psychosocial Care Centre for People with Serious Mental Disorders. Participants accessed the survey through a link created using the Google Forms tool, according to the Checklist for Reporting Results of Internet E-Surveys [20]. The data were collected from 14 April to 9 May 2021 (when the movement restrictions ended in Spain after the end of the third state of national alarm). Movement restrictions in Spain began on 14 March 2020. From that date until the time of data collection, measures of social isolation and movement restrictions continued to a greater or lesser extent throughout the three states of alarm declared in the country. Participants responded to the survey once but provided information about the study variables in reference to two moments: before and during the movement restrictions resulting from the COVID-19 pandemic. All participants entered the data in the questionnaire under the supervision of their doctor.

### 2.1. Recruitment

The inclusion criteria were institutionalised and non-institutionalised patients (male and female) with a diagnosis of schizophrenia (according to the DSM-5) who were aged over 18 years. Non-Spanish-speaking patients with difficulty understanding the Spanish language or with a diagnosis of other serious psychiatric pathologies were excluded from this study.

### 2.2. Questionnaire

The questionnaire comprised 43 items and collected information about the variables listed below.

- Sociodemographic data: sex, age, height, weight, educational level, employment situation, and type of residence (psychosocial care centre or private domicile).
- Pharmacological treatment: A specific question was performed to collected specific information on the possible change in the pharmacological treatment during the pandemic with three possible answers: increased, decreased, did not change.
- Dietary habits: using the Mediterranean Diet Adherence Screener (MEDAS) questionnaire from the PREDIMED study [21]. This questionnaire has been validated for the Spanish population and assesses adherence to the Mediterranean diet, an eating pattern which has proven to be effective in the prevention and reduction of the incidence of various diseases such as cardiovascular pathologies [21], metabolic syndrome, and type 2 diabetes [22]. This questionnaire comprises 14 items, 12 of them on the frequency of food consumption and 2 on the dietary habits characteristic of the Spanish Mediterranean diet. Each item is scored with a 0 or 1 and, based on the total score, the participants were classified as ‘low adherence’ (score between 0–5), ‘medium adherence’ (score between 6–9), or ‘high adherence’ (score ≥ 10) [14]. The good reproducibility and relative validity of this questionnaire have been evaluated elsewhere [23].
- The patient physical activity levels were assessed using the International Physical Activity Questionnaire Short Form (IPAQ-SF). This is a self-administered questionnaire comprising 7 items which collects information about the physical activity the surveyee has completed in 7 days prior. This questionnaire collects information about the days per week and minutes per day the respondents spent engaged in vigorous or moderate exercise, walking, and sedentary activities and has previously been validated by Faulkner et al. in patients with schizophrenia [24]. These authors found a correlation coefficient of 0.68 for reliability and 0.37 for criterion validity based on total reported minutes of physical activity, and concluded that, the Short-Form IPAQ, when used with individuals with schizophrenia, exhibits measurement properties that are comparable to those reported in the general population and can be considered as a surveillance tool to assess levels of physical activity. In addition, general information was collected on the obstacles to exercising perceived by the patients during the period of COVID-19-related movement restrictions.
- To assess the smoking habit of the patients, their consumption was classified into 5 categories: 0–5 cigarettes, 6–10 cigarettes, 11–15 cigarettes, 16–20 cigarettes, or more than 20 cigarettes.
- Levels of anxiety and depression were assessed using the Hospital Anxiety and Depression Scale (HADS) [25] which comprises 14 questions, of which 7 assess anxiety symptoms (HADS-A) and 7 measure symptoms of depression (HADS-D). Each item is scored from 0 to 3, with a score range on each subscale of 0–21 points. Scores of 0–7 indicate the absence of anxiety or depression; scores of 8–10 indicate mild levels; scores of 11–14 indicate moderate levels; and scores of 15–21 indicate severe levels of anxiety or depression. This scale has been validated and has adequate psychometric properties [26]; the internal consistency, as assessed by Cronbach’s alpha, was 0.90 for the full scale, 0.84 for the depression subscale and 0.85 for the anxiety subscale.

### 2.3. Ethical Considerations

This study was approved by the CEU Cardenal Herrera University Ethics Committee (CEI18/115) and followed the fundamental principles established in the Declaration of Helsinki. All participants were suitably informed about the characteristics of the study via the participant information sheet which formed the first page of the survey link. Subsequently, the patients gave their consent to participate in the study by ticking a specific box on the form. Completion of the survey was anonymous and voluntary.

### 2.4. Statistical Analysis

No prospective data are available on the symptoms of anxiety and depression among patients with schizophrenia during the movement restrictions imposed because of the COVID-19 pandemic. Thus, we hypothesised that the anxiety (HADS-A) and depression (HADS-D) symptoms of these patients would significantly increase during the pandemic. We performed a power analysis using G*Power software v3.1.9.2 (Heinrich-Heine-Universität, Düsseldorf, Germany) and found that 102 participants would provide 85% statistical power at a 5% significance level (two-sided test) for a small to medium effect size (d = 0.3).

Compliance with the assumption of normality was checked for each dependent variable by using the Kolmogorov–Smirnov test. This test indicated that the data was not normally distributed in any of the studied variables. First, we used Mann–Whitney U and Chi-squared tests to compare the values from the entire sample to check for possible differences between institutionalised and non-institutionalised patients in our cohort prior to the COVID-19 pandemic. We found no statistically significant differences between these groups for any of the studied variables, and so we pooled all these data for our subsequent analyses.

Next, we applied non-parametric Wilcoxon tests to compare values for anxiety, depression, physical activity, and eating habits before and after the COVID-19 pandemic lockdown. Chi-squared tests were also used to assess the association between categorical variables (smoking habits). The data were represented as medians and interquartile ranges (in square brackets) for continuous variables, or as a number and percentage (shown in parentheses) for categorical variables. To avoid increasing type I error by repeating the statistical tests for each of the 12 dependent variables, a Bonferroni adjustment was applied to the significance level. Thus, the alpha level for these 12 comparisons was 0.05/12, that is, *p* = 0.004. SPSS software for Windows (version 18.0, SPSS, Inc., Chicago, IL, USA) was used for all our analyses.

## 3. Results

The internet-based survey concluded on 9 May 2021; the questionnaire was sent to 110 patients with schizophrenia and a total of 103 participants (93.6%) completed it. After validation of the data, 102 respondents were finally included in the study (1 questionnaire was excluded because the survey had responded after the data collection period). The general characteristics (mean ± standard deviation) of the participants are shown in Table 1.

Regarding the pharmacological treatment, 78 of the 102 patients (76%) indicated that they had not modified their medication in relation to the pre-pandemic situation, 20 patients indicated that they had increased their consumption and 4 indicated a decrease.

Our results showed significant increases in anxiety (HADS-A), depression (HADS-D), the overall HADS score, and the number of minutes spent sitting per day, as well as significant decreases in the number of walking activities completed during the COVID-19 pandemic lockdown compared to the prior situation (Table 2). No significant changes were found in terms of vigorous and moderate activities.

Table 3 shows participant adherence to the Mediterranean diet (low, medium or high adherence).

Table 4 shows the results of the obstacles to performing physical exercise perceived by patients during the period of movement restrictions put in place as a consequence of the COVID-19 pandemic.

Table 5 shows the results from the HADS-A and HADS-D subscales according to the severity of the symptoms.

Chi-squared tests showed a significant increase in smoking habits during the movement restrictions put in place to combat COVID-19 compared to the pre-COVID-19 period (*p* < 0.001; Table 6).

## 4. Discussion

The objective of this study was to analyse the changes in the lifestyle and levels of anxiety and depression of patients with schizophrenia during the period of movement restrictions resulting from the COVID-19 pandemic lockdown compared to the time beforehand. To the best of our knowledge, this was the first study to analyse these changes in terms of diet, exercise, tobacco consumption, and levels of anxiety and depression in this specific population. The data were collected between 14 April and 9 May 2021 when the third national state of alarm ended in Spain leading to easing of the movement restrictions. The response rate to the survey used in this study was 93.6% which is comparable to that of other previous studies carried out in similar patient groups [27,28].

### 4.1. Pharmacological Treatment

Regarding the pharmacological treatment, most of the participants (76%) indicated that they had not modified their medication during the pandemic compared to the pre-pandemic situation. Only 24% of the patients indicated that they had modified their consumption. Of the patients who modified it, 19% indicated that they had increased the dose compared to 5% who indicated that they had decreased it. Our results are similar to those obtained by Mckee et al. in another retrospective observational study [29]. The authors analysed Canadian national consumption of long-acting injectable antipsychotic over different time periods during the COVID-19 pandemic. The results of their study showed that rates of the drugs prescription remained highly stable throughout the study period. On the other hand, the increase in the pharmacological treatment observed in almost 20% of the participants could be related to the increase in anxiety and depression observed during the pandemic. In fact, the results of another study carried out in the Spanish population have also shown an increase in the levels of anxiety and depression in patients with mental illness during confinement compared to the general population [17].

### 4.2. Diet

With regard to lifestyle, diet plays a fundamental role in preventing several diseases that are especially common in patients with schizophrenia, including obesity or being overweight, hypertension, and cardiovascular disease [30,31,32,33,34]. The situation produced by the COVID-19 pandemic set off alarms about the possible negative impact of social distancing measures and movement restrictions on the nutritional health of the general population. However, the results of this current study did not show significant changes in adherence to the Mediterranean diet in this population of patients with schizophrenia compared to the situation prior to the COVID-19 pandemic lockdown.

Coinciding with our results, studies carried out in other population groups also found no significant dietary changes as a consequence of the movement restrictions imposed during the COVID-19 lockdown [34,35,36]. However, when we looked at the distribution of the respondents according to the three adherence classification levels, we observed that in more than 60% of the cohort patient adherence levels to the Mediterranean diet were below the levels recommended by the Spanish Society of Community Nutrition. Specifically, the medium and lower adherence were 51% of 14.7%, respectively, while only slightly more than 30% of the participants indicated that their adherence to the Mediterranean diet was high. In a healthy general population, better results have been observed in adherence to the Mediterranean diet, with a mean adherence in 73.5% of those surveyed [37].

The poor diet quality of many of our survey participants was in line with the results from previous studies in which patients with schizophrenia consumed low amounts of fruits and vegetables [38,39,40] and had a high sugar, saturated fat, processed food, and alcohol intake [38,39,41]. The WHO definition of the overweight category is a body mass index (BMI) ≥ 25, while obesity is considered a BMI ≥ 30 [42]. The average BMI of the participants in this current work was 28.6, meaning that most of them were overweight with some values quite close to the level of obesity.

This result is especially alarming if we also consider that obesity or being overweight are risk factors for the development of cardiovascular diseases, which represent one of the main causes of premature death in patients with schizophrenia [27,31,33]. In line with this data, the results of a recent study carried out during the pandemic in patients with mental disorders found a higher body mass index compared to the general population. Furthermore, participants with mental disorders more frequently reported weight gain > 5 kg during the pandemic [43]. In addition, some authors have observed that a high BMI is a risk factor for the development of critical conditions in patients infected with SARS-CoV-2 [44], thereby representing an added risk in the population with schizophrenia.

We must also consider that more than 50% of the participants had low educational levels and that notable numbers of these patients were also unemployed (20.5%) or incapacitated for work (73.5%). Previous studies have observed that socioeconomic status was related to a poor diet in people with schizophrenia [45,46,47], which may account for the inadequate adherence to the Mediterranean diet observed in two thirds of the participants of this present work.

### 4.3. Physical Activity

During the COVID-19 pandemic, the WHO recommended regularly engaging in exercise to help promote a healthy lifestyle. Despite the known benefits of exercise in patients with schizophrenia in terms of improving cardiometabolic health [48,49], cardiorespiratory function [50], cognitive function [51], and mental health symptoms [48,49,52], under normal conditions this population is usually highly sedentary [53] or tends to engage in less vigorous or moderately vigorous exercise compared to the general population [54,55].

In this current study, 45% of the respondents did not consider exercise as a priority during the pandemic. The remaining participants indicated having encountered obstacles to engagement in exercise during the pandemic lockdown, with the main stated reasons being a lack of enthusiasm (18.6%) and the externally imposed movement restrictions (9.8%). Regarding the exercise intensity, we observed no significant differences in the levels of vigorous and moderate physical activity performed by the participants during the period of movement restrictions compared to normal situation beforehand. However, our results did show that the levels of physical activity of these patients was much lower than those recommended by the WHO which established a weekly minimum of 75 min of vigorous exercise or 150 min of moderate exercise, or a combination of both [56].

Specifically, the patients included in our cohort had dedicated less than one day a week to vigorous or moderate exercise, with a mean of 12.9 and 17.9 min/week for vigorous and moderate exercise, respectively. In turn, these results were accompanied by a significant decrease in the time the participants had spent walking and a concurrent increase in the time they had spent engaged in sedentary activities. These individuals had gone from walking an average of 330 min per week to only 86 min per week, representing an almost 75% decrease.

This data is especially worrisome if we also consider that walking is usually the most common type of physical activity these patients perform [57]. However, this striking 75% reduction in the time spent walking may have been because this was their main means of transportation [58] which consequently limited their movements after the measures meant to contain the spread of SARSCoV2 were imposed. This may have been compounded by the significant increase in the time this group had spent engaged in sedentary activities, which went from 282 min prior to the lockdown to 398 min during the COVID-19 pandemic.

In the healthy population, a general decrease in physical activity levels has also been observed as a consequence of confinement. Specifically, the results of a study carried out on 3.800 Spaniards showed a decrease in the levels of vigorous physical activity and in the time spent walking, and an increase in sedentary lifestyle. In our study, in contrast with the results observed in the general population, the participants did not show a decrease in the level of vigorous physical activity [59]. However, as mentioned above, it must be borne in mind that we are referring to a population group that under normal conditions is already very sedentary in itself and practices less physical exercise compared to healthy people [54,55].

### 4.4. Tobacco Consumption

Tobacco consumption is another unhealthy habit that people with schizophrenia frequently engage in, and indeed, this factor was one of the main risk factors for premature death in this population group [60]. Previous studies place the prevalence of smoking in these patients at 50–90%, depending on the country considered [61,62,63]. Additionally, there is a strong link between smoking and schizophrenia [63,64], with a large proportion of these individuals being chain smokers who show serious difficulties in quitting smoking, both in the short and long term [65].

The results of this study showed that 59.8% of the respondents were smokers. This data coincides with the prevalence percentages of previous studies in smokers with schizophrenia in Spain, with values of 53–69% [64,66,67]. Moreover, our results showed a significant increase in the tobacco consumption of the participants during the COVID-19 pandemic lockdown period. Almost half of the smokers in our cohort (49.1%) indicated that they had increased their consumption compared to the pre-pandemic situation, while only 8.8% indicated that they had decreased their consumption during the same period. These results are similar to those reported by Peckham et al. [68] who analysed the smoking habits of 367 people with severe mental illness during the COVID-19 pandemic lockdown. Their results showed an increase in tobacco consumption in 54.5% of those surveyed, and a decrease in 12.1%.

### 4.5. Anxiety and Depression

In terms of anxiety and depression, the participants showed a significant increase in the scores obtained for the two HADS subscales during the COVID-19 pandemic movement restrictions compared to the previous situation. Around 65% of the patients had shown symptoms of mild, moderate, or severe anxiety during the pandemic, compared to 42% beforehand. In addition, the number of patients with moderate symptoms almost doubled and this increase was even more striking (17-fold higher) among patients who showed severe symptoms of anxiety. The number of participants who showed symptoms of depression was also similar to the figures recorded for anxiety at 62.7% during the lockdown compared to 40.1% prior to the COVID-19 pandemic lockdown. Furthermore, more participants began to show moderate to severe symptoms of depression compared to the situation prior to the lockdown.

These results are similar to those observed in a cross-sectional study carried out in China during the COVID-19 pandemic [28]. These authors analysed the symptoms of anxiety and depression in 1063 patients with psychiatric disorders and found a prevalence of 52.4% and 62.3% for anxiety and depression, respectively. In addition to coinciding with our results, Solé et al. [17] observed higher levels of anxiety and depression in 206 Spanish patients with psychiatric disorders, including those with schizophrenia, as a consequence of the movement restrictions imposed, compared to the 413 control group participants. Nevertheless, none of these studies specifically analysed patients with schizophrenia.

Therefore, the results of this study provide novel data on the effects of the movement restrictions imposed as a consequence of the COVID-19 pandemic on the lifestyle of patients with schizophrenia. Despite the WHO [1] recommendations designed to help people maintain a healthy lifestyle during the pandemic, these lifestyle parameters worsened in the patients included in this current study because they had spent more time engaged in sedentary activities, less time walking, and had increased their tobacco use. These findings, in addition to the fact that many of these individuals already had an inadequate diet, highlights the need to implement programs specifically aimed at this patient type to promote healthy lifestyles and behaviours and help reduce the morbidity and mortality associated with schizophrenia, especially in possible similar future situations involving movement restrictions.

In this sense, studies carried out in other population groups such as in obese patients, have shown that online interventions have the same efficacy as traditional ones [69,70]. Recently, preliminary results have also been published on the efficacy of online interventions in patients with schizophrenia [71]. In addition, such online programmes have the advantage that they can be self-administered by the patients themselves, making them potentially useful tools in possible future pandemic situations.

This study had some limitations including recall bias (responses conditioned by the recall capacity of the participants) and social desirability bias (a tendency to exaggerate healthy habits and minimise harmful ones) [72]. Furthermore, the sample was not homogeneous because we included both institutionalised and non-institutionalised patients. Nonetheless, the results of our statistical analyses did not show any significant differences between these two patient sub-groups. Finally, we only collected information about possible changes in drug use during the pandemic, but we did not quantify the equivalent dose of chlorpromazine of the patients.

## 5. Conclusions

During COVID-19 pandemic decreased the time patients with schizophrenia spent walking and increased the number of hours they spent engaged in sedentary activities, as well as both their tobacco consumption and their levels of anxiety and depression. However, their adherence to the Mediterranean diet, as well as the levels of moderate to vigorous physical exercise they performed, had remained unaltered. Notwithstanding, both the quality of their diets and the levels of physical exercise they engaged in were below the levels considered healthy according to the WHO. The results of this study reveal the importance of developing specific interventions, especially those based on the internet due to their easy access, that can promote healthy lifestyle habits in patients with schizophrenia in similar future situations.

## Figures and Tables

**Table 1:** The general characteristics of the participants before the COVID-19-related lockdown.

	Institutionalised (*n* = 64)	Non-Institutionalised (*n* = 38)	All Patients (*n* = 102)
Age (years)	45.7 (11.1)	45.1 (13)	45.5 (11.8)
Weight (kg)	78.7 (15.8)	86.4 (18.4)	81.6 (17.1)
Height (cm)	168 (9)	170 (9)	169 (8.6)
Body mass index (kg/m^2^)	27.8 (5)	29.9 (6.7)	28.6 (5.7)
Female/Male (n)	24/40	9/29	33/69
**Educational level**			
Low	43 (42.1)	13 (12.7)	56 (54.9)
Intermediate	18 (17.6)	20 (19.6)	38 (37.2)
High	3 (2.9)	5 (4.9)	8 (7.8)
**Employment situation**			
Work incapacity	51 (78.5)	24 (63.2)	75 (73.5)
Employed	2 (3)	2 (5.2)	3 (2.9)
Unemployed	11 (16.9)	10 (26.3)	21 (20.5)
Studying	1 (1.5)	2 (5.2)	3 (2.9)

These values are expressed as the mean (SD) for age, weight, height and body mass index, and as number (%) for educational level and employment situation.

**Table 2:** Results of the non-parametric Wilcoxon signed-rank tests.

			Before vs. during the COVID-19 Lockdown	
VARIABLES	Pre-COVID-19	During-COVID-19	*p*	*Z*
HADS-Anxiety	7 (4)	9 (6)	<0.001 *	−7.45
HADS-Depression	6 (7)	9 (6.3)	<0.001 *	−7.03
HADS-Total	14 (10)	17.5 (11)	<0.001 *	−7.76
MEDAS	9 (3.3)	9 (3.5)	0.444	−0.77
Vigorous activity (days per week)	0 (0)	0 (0)	0.605	−0.52
Vigorous activity (min per day)	0 (0)	0 (0)	0.082	−1.74
Moderate activity (days per week)	0 (1)	0 (0)	0.117	−1.57
Moderate activity (min per day)	0 (60)	0 (0)	0.018	−2.37
Walking activity (days per week)	5 (5)	0 (7)	<0.001 *	−6.36
Walking activity (min per day)	60 (42.5)	0 (60)	<0.001 *	−6.32
Sitting (min per day)	240 (120)	360 (240)	<0.001 *	−6.73

These values are expressed as medians and interquartile ranges (in parentheses); * *p* < 0.004; Z-scores for the Wilcoxon signed-rank test.

**Table 3:** Adherence to the MD.

Adherence to the MD	Pre-COVID-19	During COVID-19
LOW	15 (14.7)	16 (15.7)
MEDIUM	52 (50.9)	48 (47.0)
HIGH	35 (34.3)	38 (37.2)

Data are expressed as number and a percentage in parenthesis [*n* (%)].

**Table 4:** Perceived obstacles to exercising during the COVID-19 pandemic lockdown.

None	22 (21.6)
Not being in the mood	19 (18.6)
Not a priority	46 (45)
Not being able to leave home	10 (9.8)
Others	5 (4.9)

These values are expressed as a number and a percentage (shown in parentheses).

**Table 5:** Categorisation of anxiety and depression levels according to severity of the reported symptoms.

	Pre-COVID-19	During the COVID-19 Lockdown
**Anxiety**		
No anxiety	59 (57.9)	35 (34.3)
Mild	25 (24.5)	24 (23.5)
Moderate	17 (16.7)	26 (25.5)
Severe	1 (0.9)	17 (16.7)
**Depression**		
No depression	61 (59.8)	38 (37.2)
Mild	28 (27.4)	29 (28.4)
Moderate	11 (10.8)	25 (24.5)
Severe	2 (1.9)	10 (9.8)

These values are expressed as a number and a percentage (shown in parentheses).

**Table 6:** Results for the changes in the patient smoking habits during the COVID-19 pandemic lockdown.

VARIABLES	Pre-COVID-19	During the COVID-19 Lockdown
0–5 cigarettes per day	43 (42.2)	43 (42.2)
6–10 cigarettes per day	20 (19.6)	15 (14.7)
11–15 cigarettes per day	13 (12.7)	14 (13.7)
16–20 cigarettes per day	21 (20.6)	19 (18.6)
More than 20 cigarettes per day	5 (4.9)	11 (10.8)

These values are expressed as a number and a percentage (shown in parentheses).

## Data Availability

The original contributions presented in the study are included in the article, further inquiries can be directed to the corresponding author.
